# Successful surgical repair of adult anomalous origin of the left main coronary artery from the pulmonary artery syndrome complicated by severe mitral regurgitation: a case report

**DOI:** 10.1093/ehjcr/ytad340

**Published:** 2023-07-21

**Authors:** Tetsuya Saito, Masashi Kawamura, Koichi Toda, Shigeru Miyagawa

**Affiliations:** Department of Cardiovascular Surgery, Osaka University Graduate School of Medicine, 2-2 Yamadaoka, Suita, Osaka, Japan, 565-0871; Department of Cardiovascular Surgery, Osaka University Graduate School of Medicine, 2-2 Yamadaoka, Suita, Osaka, Japan, 565-0871; Department of Cardiovascular Surgery, Osaka University Graduate School of Medicine, 2-2 Yamadaoka, Suita, Osaka, Japan, 565-0871; Department of Cardiovascular Surgery, Osaka University Graduate School of Medicine, 2-2 Yamadaoka, Suita, Osaka, Japan, 565-0871

**Keywords:** Anomalous origin of the left main coronary artery from pulmonary artery, Mitral regurgitation, Atrial fibrillation, Mitral valve repair, Case report

## Abstract

**Background:**

The number of diagnosed cases of anomalous origin of the left main coronary artery from the pulmonary artery (ALCAPA) in adults has increased substantially because of modern advances in non-invasive cardiac imaging. Here, we report successful surgical repair in an adult patient with ALCAPA complicated by severe mitral regurgitation (MR) and persistent atrial fibrillation.

**Case summary:**

ALCAPA syndrome was detected in a 65-year-old Asian woman with persistent atrial fibrillation by coronary computed tomographic angiography. An echocardiogram revealed severe MR caused by annular dilation, atrial enlargement, and posterior mitral leaflet tethering. In addition to ALCAPA repair, mitral valve repair and Cox-Maze IV cryoablation were performed. Mitral valve repair was performed using augmentation with an autologous pericardial patch in the posterior leaflet and ring annuloplasty.

**Discussion:**

Because the mechanism of MR with ALCAPA in an adult varies by comorbidity, mitral valve repair should be performed according to the valvular and subvalvular morphologies. It is essential to develop strategies that provide adequate myocardial protection during the surgical treatment of ALCAPA considering coronary steal and non-coronary collateral blood flow.

**Learning points**

1. To be familiar with the presence and clinical course of ALCAPA in adults, their comorbidities, and treatment indication.
2. To gain knowledge about the mechanisms of mitral regurgitation in adult patients with ALCAPA and treatment strategies for mitral valvuloplasty according to valvular and subvalvular morphologies.
3. To develop strategies that provide adequate myocardial protection during the surgical treatment of ALCAPA considering coronary steal and non-coronary collateral blood flow.

## Introduction

Anomalous origin of the left coronary artery from the pulmonary artery (ALCAPA) is a rare congenital abnormality associated with early infant mortality and sudden death in adults. The incidence of ALCAPA is estimated to be 1 per 300 000 live births, and this condition accounts for 0.24–0.46% of all congenital cardiac diseases.^1^ ALCAPA can be classified into infantile (early presentation) and adult (late presentation) types. The nature and timing of ALCAPA presentation may vary depending on the adequacy of the collateralized left coronary artery (LCA) circulation. Adult-type ALCAPA presents with significant collaterals to the left coronary artery. Even with the presence of collateral vessels, chronic sub-endocardial ischaemia occurs in most cases and patients die from sudden cardiac death. Adult ALCAPA patients can present with various symptoms, including atrial fibrillation, dilated cardiomyopathy, mitral regurgitation (MR), acute myocardial infarction, angina, and dyspnoea on exertion.^2^ The number of diagnosed cases of ALCAPA in adults has increased substantially related to modern advances in non-invasive cardiac imaging.^3,4^

The European Society of Cardiology adult congenital heart disease (ESC ACHD) clinical practice guidelines recommend surgical repair for adult patients with ALCAPA.^5^ However, there are no fixed guidelines for the management of MR in adult patients with ALCAPA. Here, we report successful surgical repair in an adult patient with ALCAPA with severe MR and persistent atrial fibrillation.

## Summary figure

**Figure:**
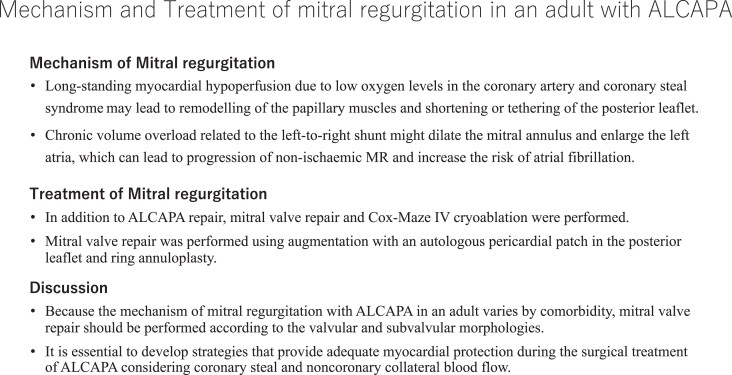


## Case summary

A 65-year-old Asian woman with palpitations and mild dyspnoea on exertion was planned to receive ablation therapy for persistent atrial fibrillation. The patient was healthy and had no symptoms of heart failure during her pregnancy at the age of 30. However, she had hypertension and developed paroxysmal atrial fibrillation after undergoing breast cancer surgery at the age of 63; conservative management was pursued. At the age of 65, she experienced worsening dyspnoea on exertion and became aware of palpitations during atrial fibrillation, which recurred despite treatment with verapamil and pilsicainide, a class Ic anti-arrhythmic drug.

**Figure 1:**
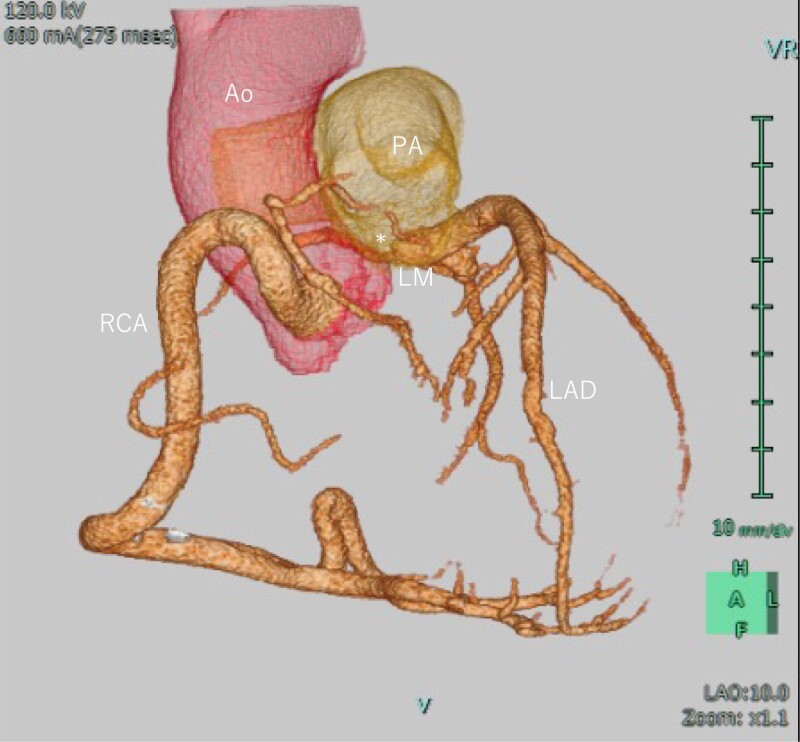
Pre-operative coronary computed tomographic angiography (CTA). The dilated RCA arising normally from the aorta and the left main coronary artery (LMCA) abnormally originating from the main pulmonary artery.

**Figure 2:**
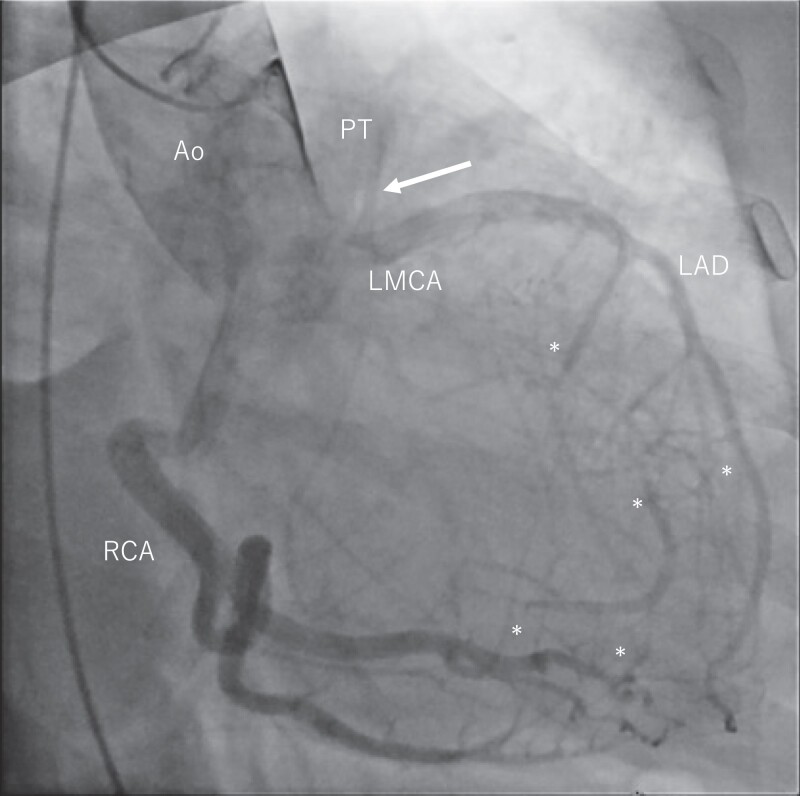
Coronary angiography of the RCA showing the dilated RCA and enlarged extensive collateral supply to the left coronary artery (LCA). Continuous arterial flow from the LCA into the pulmonary trunk is shown. This finding represents the ‘steal phenomenon’ (arrow), the main diagnostic feature of ALCAPA.

**Figure 3:**
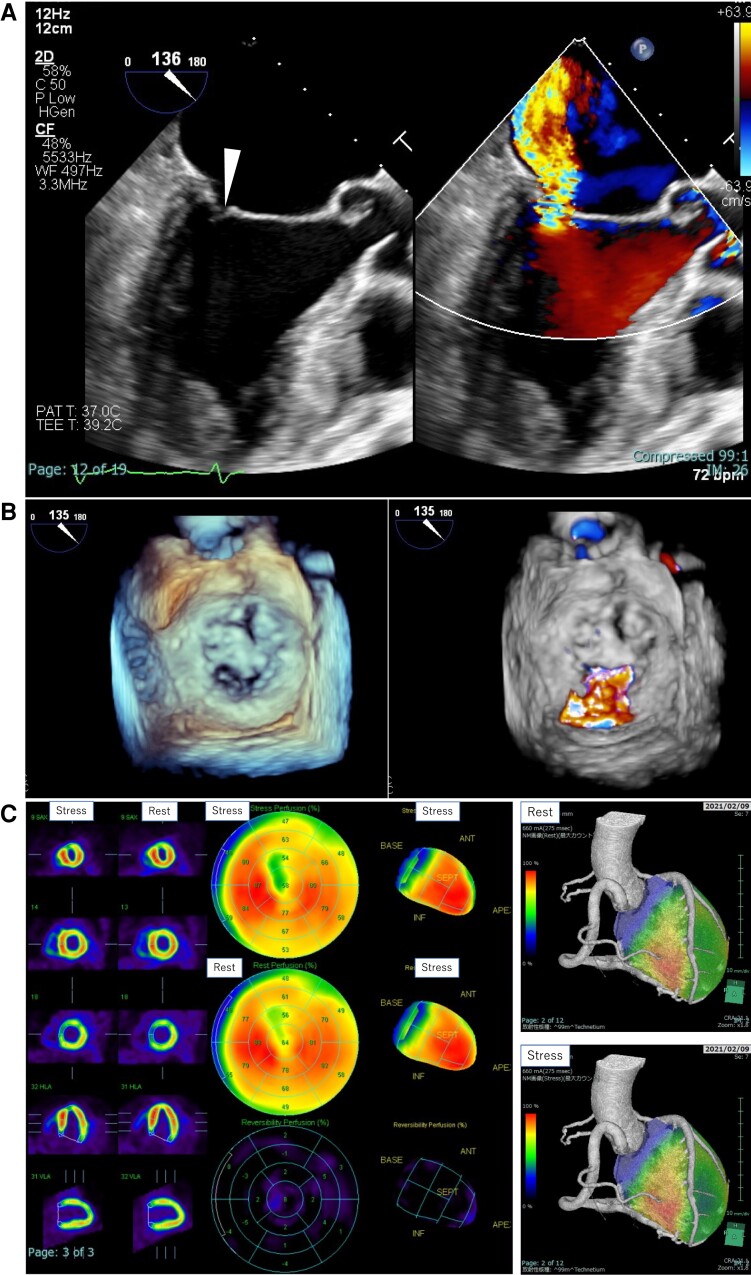
Pre-operative mitral regurgitation and myocardial perfusion. (*A*) Pre-operative transoesophageal echocardiography: long-axis view in mid-systole. Mitral annular dilatation and LA dilatation are shown. The shortened and tethered posterior leaflet and pseudo-prolapse of the anterior leaflet are shown. The arrowhead indicates the pseudo-prolapse of the anterior leaflet. (*B*) Three-dimensional (3D) echocardiography. Transoesophageal echocardiography shows a shortened and tethered mitral posterior leaflet and severe mitral regurgitation. (*C*) 99mTc-MIBI stress–rest myocardial perfusion scintigraphy. Exercise stress myocardial scintigraphy showed decreased accumulation in the antero basal-mid portion and apex, at rest and during exercise, with no myocardial ischaemia.

Physical examination revealed clear respiratory sounds and a Levine 2/6 systolic murmur without leg oedema. The patient had mild shortness of breath on daily exertion and her functional status was New York Heart Association (NYHA) class 2. The chest X-ray showed cardiomegaly (cardiothoracic ratio (CTR) 58%] without pulmonary congestion. She was taking anti-coagulation, anti-arrhythmic, and anti-hypertensive drugs consisting of edoxaban 30 mg, bisoprolol fumarate 2.5 mg, pilsicainide 150 mg, verapamil 40 mg, amlodipine 2.5 mg, and candesartan 4 mg. Transthoracic echocardiogram (TTE) in sinus rhythm showed preserved left ventricular systolic function [left ventricular ejection fraction (LVEF) 56%], mild to moderate MR [vena contracta (VC) 4.5 mm, effective regurgitant orifice area (EROA) 0.19 cm², and regurgitant volume (RV) 28 mL], and an enlarged left atrium [left atrial volume index (LAVI) 78 mL/m²]. A coronary artery–pulmonary artery (PA) fistula was detected as continuous flow into the PA with diastolic predominance. However, pre-procedural coronary computed tomographic angiography incidentally revealed the presence of a LCA abnormality originating from the pulmonary trunk (*Figure 1*). Coronary angiography revealed a dilated right coronary artery (RCA) and enlarged extensive collateral supply to the LCA. Furthermore, continuous arterial flow from the LCA into the PA was observed (*Figure 2*). Moreover, pre-operative TTE in sinus rhythm demonstrated normal left ventricular systolic function (LVEF 62%) and moderate MR [VC 5.5 mm, EROA 0.29 cm^2^, RV 58 mL, and regurgitant fraction (RF) 55%] related to annular dilation [mitral annulus anteroposterior diameter (AP) 35 mm] along with left atrial enlargement (LAVI 98 mL/m^2^). Transoesophageal echocardiography (TEE) showed a dilated mitral annulus [AP 34 mm; intercommissural diameter (CC) 30 mm] and a shortened and severely tethered posterior leaflet (*Figure 3A* and *B*), indicating MR Carpentier type IIIb. Exercise stress myocardial scintigraphy showed decreased accumulation in the anterior wall mid portion–apex at rest and during exercise, with no signs of myocardial ischaemia (*Figure 3C*). Therefore, the patient was diagnosed with ALCAPA complicated with symptomatic MR and persistent atrial fibrillation.

**Figure 4:**
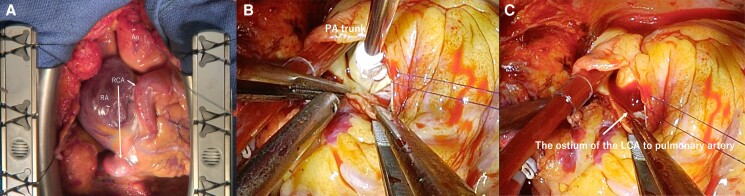
Intra-operative findings of ALCAPA. (*A*) Intra-operative inspection of the huge right coronary artery (arrow). (*B*) Administration of selective cardioplegia through the ostium of the left coronary artery originating from the left posterior sinus of the pulmonary artery. (*C*) Non-coronary collateral blood flow (NCCF) was confirmed by the continuous blood flow from the ostium of the LCA to the pulmonary artery despite cardioplegia and lack of coronary circulation.

**Figure 5:**
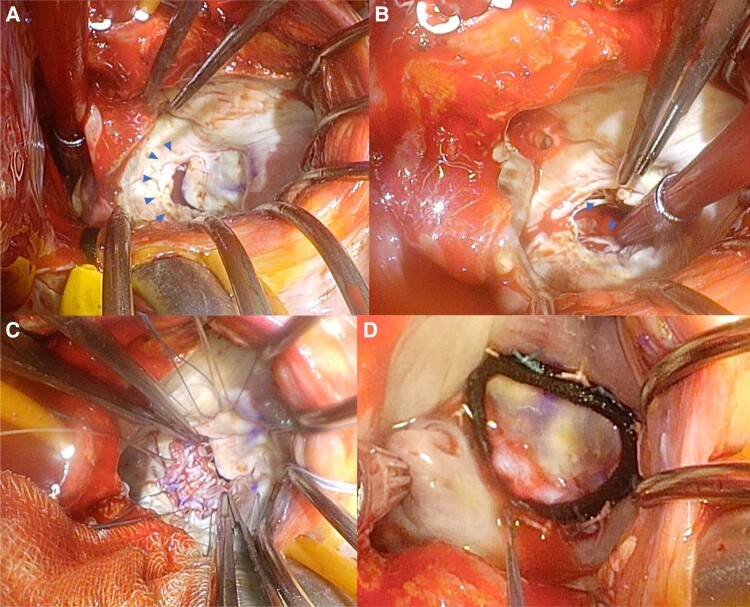
Intra-operative findings of mitral valvular and subvalvular morphologies and mitral valve plasty. (*A*) Shortened posterior mitral leaflet and the gap between the anterior and posterior leaflets. (*B*) No operative finding of fibrosis or degenerating papillary muscles. (*C*) Patch augmentation of the posterior mitral leaflet with fresh autologous pericardium. (*D*) The final test with saline demonstrates a perfectly competent mitral valve.

We performed ALCAPA repair, mitral valve repair, and Cox-Maze IV cryoablation. Following median sternotomy, cardiopulmonary bypass was initiated with aortic and bicaval venous cannulation. Surgical revascularization of the saphenous vein to the left anterior descending artery was performed in an on-pump beating fashion. Cardiac arrest was achieved through antegrade cold blood cardioplegia, which simultaneously occluded the ostium of the LCA after opening of the pulmonary trunk. We added selective cardioplegia through the ostium of the LCA originating from the left posterior sinus of the PA (*Figure 4A*). Non-coronary collateral blood flow (NCCF) was confirmed by the continuous blood flow from the ostium of the LCA into the PA during cardiac arrest without the infusion of cardioplegia (*Figure 4B*). We occluded the ostium of the LCA in the PA during the additional infusion of antegrade cardioplegia. Subsequently, we opened the ostium for systemic collateral flow drainage to the PA during cardiac arrest to maintain myocardial protection by reducing cardioplegia wash out in the collateral flow territory. Cox-Maze IV cryoablation was performed, the mitral annulus was dilated, and the posterior mitral leaflet was shortened (P2: 9 mm) and severely tethered to the left ventricle (*Figure 5A*). There was no apparent fibrosis or degeneration of the papillary muscle (*Figure 5B*). We performed autologous fresh pericardium patch augmentation of the posterior leaflet and mitral valve annuloplasty using MEMO 3D 30 mm (CORCYM, London, UK) (*Figure 5C* and *D*). Finally, we conducted the direct closure of the ostium of the LCA via mattress sutures and autologous pericardial pledgets.

The post-operative course was uneventful, and TTE before discharge revealed trivial MR and decreased left atrial volume (LAVI 54.1 mL/m^2^). Paroxysmal atrial fibrillation was observed during the first post-operative month, and atrial fibrillation did not recur after taking bisoprolol 2.5 mg, amiodarone 100 mg, and enalapril 2.5 mg, leading to the discontinuation of amiodarone 1 year after surgery. At 1 year follow-up, despite slight dyspnoea on exertion, the patient had a sinus rhythm, improved left ventricular systolic function (LVEF 70%), reduced left atrial volume (LAVI 62 mL/m^2^), and mild MR on TTE.

## Discussion

ALCAPA is a rare congenital abnormality^6^ causing a left-to-right shunt, leading to coronary steal, impaired myocardial perfusion, and volume overload. In adults with ALCAPA, collateral blood flow from the RCA to the LCA and NCCF contributes to survival beyond childhood.^7^ Chronic unattended ALCAPA in adults results in low oxygen levels in the coronary artery, coronary steal syndrome, and myocardial ischaemia. ALCAPA can present as a silent or symptomatic myocardial infarction, LV dysfunction, VTs, or even sudden cardiac death. Patients with ALCAPA may also primarily present with volume overload due to L-R shunt causing heart failure symptoms, MR, and atrial fibrillation.

Mitral regurgitation is a major concern in adult patients with ALCAPA. The mechanism underlying MR in ALCAPA is primarily associated with ischaemic damage to the papillary muscle and left ventricular remodelling following ischaemic dilatation, especially in infants.^8^ Although there was no intra-operative finding of fibrosis or degenerating papillary muscle, pre-operative scintigraphy revealed myocardial hypoperfusion without myocardial ischaemia. Long-standing myocardial hypoperfusion may lead to remodelling of the papillary muscles and shortening or tethering of the posterior leaflet. Furthermore, chronic volume overload related to the left-to-right shunt might dilate the mitral annulus and enlarge the left atria, which can lead to progression of non-ischaemic MR and increase the risk of atrial fibrillation.

There are no fixed guidelines for the management of MR in adult patients with ALCAPA. In infants, functional MR can improve significantly after coronary revascularization without simultaneous MV repair if there are no structural problems with the MV.^9^ Nevertheless, since chronic cardiac injury can persist after surgery,^8,10^ MV repair is recommended for adult-type ALCAPA with moderate–severe MR or pre-operative valvular configuration abnormalities. Moreover, Li et al.^11^ reported that, although post-operative echocardiography showed some residual post-operative damage within the papillary muscle, there was notable MR improvement and alleviation of LV and LA dilation after concomitant MV repair in adult patients with ALCAPA. In our patient, because of restricted leaflet motion caused by posterior leaflet tethering, mitral annuloplasty alone did not constitute an appropriate coaptation area to control MR. Therefore, we successfully performed patch augmentation of the shortened mitral posterior leaflet with fresh autologous pericardium (FAP) to secure a sufficient coaptation area and ring annuloplasty. Patch augmentation of the mitral valve with autologous pericardium was used for infective endocarditis, rheumatic mitral disease, and atrial functional MR.^12^ Fresh autologous pericardium is readily available, is non-immunogenic, has native valve tissue characteristics, has long-term durability, and presents low risk for thrombogenicity. Quinn et al.^13^ reported good long-term results with FAP augmentation without evidence of late patch calcification, stiffness, or aneurysmal degeneration. As the mechanism of MR with ALCAPA in adults varies, mitral valve repair should be performed according to valvular and subvalvular morphologies.

Currently, the dual coronary system used to repair ALCAPA in adults^14^ uses various techniques including ligation of the anomalous left main coronary artery (LMCA) in combination with coronary artery bypass graft (CABG), Takeuchi procedure, or direct implantation.^14–16^ In our patient, the implantation of LMCA was challenging because of calcification. Thus, we ligated the anomalous LMCA and performed CABG to the left anterior descending artery with a saphenous vein graft, not the internal thoracic artery, to prevent calibre mismatch and competitive flow.

Due to the prolonged duration of cardiac arrest during concomitant ALCAPA and mitral valve surgery, ensuring adequate myocardial protection with the optimal administration of cardioplegia is crucial. A previous study reported patients with ALCAPA had NCCF (bronchial artery)^17^ to the coronary artery, which washed out the cardioplegia during cardiac arrest. Regarding myocardial protection for patients with ALCAPA, we always attempt to prevent myocardial ischaemia secondary to the stealing of coronary blood flow into the PA and reduce cardioplegia washout in the NCCF territory during cardiac arrest. In our patient, there was no ventricular fibrillation or beating during cardiac arrest. If cardioplegia is inadequate, systemic hypothermia and hyperkalaemia may be alternatives for better myocardial protection.^18^

## Conclusion

We conducted successful surgical repair in an adult patient with ALCAPA syndrome complicated with severe MR and persistent atrial fibrillation. Since the mechanism of MR with ALCAPA in adults varies related to comorbidities, mitral valve repair should be performed according to valvular and subvalvular morphologies.

## Data Availability

No new data were created or analyzed in this article.

## Lead author biography

Dr Saito graduated from Osaka University School of Medicine in 2003. He is working at Osaka University, Department of Cardiothoracic Surgery. His speciality is adult congenital surgery, valvular disease, coronary artery disease, and mechanical circulatory support.
